# The Care of Our Hybrid Selves: Ethics in Times of Technical Mediation

**DOI:** 10.1007/s10699-015-9440-0

**Published:** 2015-10-29

**Authors:** Steven Dorrestijn

**Affiliations:** Hospitality Business School, Saxion University of Applied Sciences, Handelskade 75, Postbus 501, 7400 AM Deventer, Netherlands

**Keywords:** Philosophy of technology, Technical mediation, Art of living, Care of the self, Michel Foucault

## Abstract

What can the art of living after Foucault contribute to ethics in relation to the mediation of human existence by technology? To develop the relation between technical mediation and ethics, firstly the theme of technical mediation is elaborated in line with Foucault’s notion of ethical problematization. Every view of what technology does to us at the same time expresses an ethical concern about technology. The contemporary conception of technical mediation tends towards the acknowledgement of ongoing hybridization, not ultimately good or bad but ambivalent, which means for us the challenge of taking care of ourselves as hybrid beings. Secondly, the work of Foucault provides elements for imagining this care for our hybrid selves, notably his notions of freedom as a practice and of the care of the self. A conclusions about technical mediation and ethics is that whereas the approaches of the delegation of morality to technology by Latour and mediated morality by Verbeek see technical mediation of behavior and moral outlook as an answer in ethics, this should rather be considered the problem that ethics is about.

## Introduction

In the recent revival in philosophy of the art of living, Michel Foucault’s later work has played an important role. This essay explores how Foucault’s work on ethics as art of living can help define new perspectives in the ethics of technology.

The background of this question is that in our so-called postmodern time the understanding of ethics has become troubled. In modern philosophy ethics circled around the notion of laws or rational principles coupled with the notion of humans as free, autonomous agents (able to respond to the demands of morality). In postmodern thought the focus has shifted to the ways in which language, social structures and power as well as the material surroundings condition the existence of people. The philosophy of technology played a key role in this debunking of long-held suppositions about the moral subject. This is not surprising. For, philosophy of technology is about the character of technology and how technology shapes human culture and behaviors.

For thinkers about technology in the twentieth century such as Heidegger, Ellul or Mumford, revealing the social effects of technology served a critical purpose; their writings were clearly “warning calls” against the rushing spread of technology. Recent philosophy of technology rather emphasizes the fact that human existence is always, and inescapably, marked and influenced by technology. In the current terminology: “technical mediation” is all around. This view has on the one hand stimulated more practice and application oriented research. On the other hand, the inescapability of the effects of technology seems to dissolve the ground for a more critical, ethical stance. That made Langdon Winner (1993) worry that research on technology had been emptied of its critical spirit.

The question concerning technology and ethics in our postmodern situation is therefore if and how acknowledgment of mediation can go together with an ethical analysis. Two scholars who explicitly addressed this question are Bruno Latour and Peter-Paul Verbeek. Morality is not a purely human affair but often “delegated to things”, analyzed Latour (1992), and Verbeek concluded that “morality is mediated” (2011). These answers are not yet altogether adequate and satisfying. The message that ethics cannot neglect the importance of technology is clear and well taken. But “how is mediated morality different from a reduction of ethics to the rule of technology?” is the obvious and pertinent follow up question.

In *Moralicide* (‘the extinction of morality’) Huijer and Smits (2010) doubt that ethics will survive the mediation approach, unless ‘new ethical vocabularies’ are further elaborated. As a contribution to the search for new perspectives in ethics which suit contemporary conceptions of technical mediation, I will explore what Michel Foucault’s work, especially his turn to ethics as art of living has to offer. Not only will I employ Foucault’s work to find new directions for ethics in relation to technology. I will also, to begin with, explore the meaning of “technical mediation”, the ethical “trouble maker”. That analysis is equally inspired by Foucault and prepares the way for the presentation of how an art of living with technology can contribute to “ethics in times of technical mediation”.

## Figures of Technical Mediation

While the term “technical mediation” has been often employed (McLuhan 2003; Ihde 1990; Feenberg 2002; Latour 1999; Kockelkoren 2003), notably Peter-Paul Verbeek (2005; 2011) has adopted it as the central concept in his philosophy of technology, advancing towards a “theory of technical mediation”. I will also adopt a technical mediation approach, but with the important amendment that it does not necessarily denote the latest *theory* but rather an enduring *theme* or *problem*.

According to Verbeek, technical mediation theory can offer an alternative to the dominant critique of technology as a dangerous power opposing humanity, without falling back into the naïve notion of technology as neutral instruments. For Verbeek the dystopian critique of technology can be countered by a better theory of humans and technology. Technical mediation theory could substitute inadequate understandings which are based on the separation between instead of on the entwinement of humans and technology.

To start with, the analysis that earlier philosophies of technology lacked a profound understanding of the entanglement of human existence with technology in terms of mediation, can be contested. Rather, the very different ethical evaluation of the mediation by technology marks the difference between contemporary and earlier currents in the philosophy of technology. I do not think that finding the adequate theory leads to adequate ethical views and can take away misplaced fears of technology.

It is true that the specific account of how technology mediates human existence is reflected by a specific ethical evaluation. But the order is not fixed. Worries about technologies feed theories about technology, as much as understandings of technologies inform ethical evaluations. Technical mediation then denotes the *theme* of the mutual dependency of humans and technology. It is not an *answer* (the latest and most adequate theory), but it refers to a *problem*, namely the question of how our existence is entangled in technology. This take on technical mediation is inspired by Michel Foucault, for whom seeking understanding of ourselves and our situation cannot be separated from ethical concern. In his late work Foucault speaks of “ethical problematization” (Foucault 1992) and of a “critical attitude” (Foucault 2000a, 319), both denoting a simultaneous “analysis of the limits imposed on us and an experiment with the possibility of going beyond them” (ibid.).

So, while technical mediation may not have been a key concept in earlier phases of the philosophy of technology, it is still possible to ask which “figures of technical mediation” have been discovered. The term can serve as a lens through which one can give a new account of past findings in the light of a philosophy of technology centered on the notion of technical mediation. By looking for figures of technical mediation and correlating figures of ethical concern, I will sketch an historical background that will help to understand today’s challenges in the ethics of technology.

### Fabulous but Scarce Technology

In ‘early philosophy of technology’, the first phase of philosophical thinking about technology, from the Enlightenment until well into the twentieth century, the dominant conception of technology was, in general, very positive, sometimes ‘utopian’. Scientific reason and technical progress would bring humanity to a next stage, progressively overcoming the precarious state of human existence, thus moving towards perfection and completion. Scarcity and unequal distribution of technology were the only hindrances to the full benefit of the wonders of technology.

Ernst Kapp and Karl Marx are two relevant thinkers with respect to the early philosophy of technology. Both of them followed up on the philosophy of Hegel who conceived of human history as the ongoing process in the direction of complete self-consciousness. As awareness of the determining forces of nature and society grows, human consciousness at the same time detaches itself from these determinations. This is the well-known dialectical scheme in Hegel’s philosophy: a kind of zigzag movement towards ever more complete consciousness and freedom. Both Kapp and Marx employ this scheme for understanding the relation between technology and humans.

Ernst Kapp, in his book *Grundlinien einer Philosophie der Technik* (1877),^1^ seems to have been the first to explicitly use the phrase ‘philosophy of technology’. Kapp was interested in understanding what technology is and how it develops. For this he employed the dialectical scheme. He asserted, firstly, that al technologies are projections of human organs. Whether or not human inventors are aware of it, all technologies, in Kapp’s understanding, are exteriorizations of functions of the human body. The hammer extends the fist; the wheel is an extension of the human walking movement; the telegraph is a projection of the nervous system, et cetera. This is step one in the zigzag movement. To this, secondly, Kapp adds that in a return movement, humans start understanding themselves as mechanisms. Man only gains self-understanding after he has reproduced himself in technological extensions: the skeleton came to be seen as a mechanism; the heart was defined as a pump. Concurrently with their technical activities humans gain more complete self-understanding.

Early philosophy of technology discovered how technology mediates human existence along the lines of the fairly broad and abstract idea that *the completion of human existence is interwoven with and achieved by means of the development of technology*. When halfway the twentieth century (in 1946/1947) French philosopher Georges Canguilhem was commenting on ideas like that of Kapp, he asserted that this view of technology implies that it is a matter of course that “machine” and “organism” will proceed to merge, thereby mutually contributing to the completion or perfection of both. Only in the very last sentences Canguilhem remarks that to ask whether this development is ethically desirable, would be ‘still an altogether different question’ (Canguilhem 1965, 127; my transl.) The early approach to technology was more focused on what technology is and how it develops than on ethical evaluation.

In the same period of early philosophy of technology, Karl Marx, too, employed a dialectical scheme for describing historical progress. Whereas for Hegel the ongoing development of self-consciousness was ‘pulling’ history, Marx turned this upside down and found that it were the historical conditions that ‘pushed’ the development of the spirit. ‘Life is not determined by consciousness, but consciousness by life’ (Marx and Engels 1970: 47). The material-economic basis, which refers to the availability of resources and means of production is the condition of consciousness, spiritual life. The material-economic conditions have a clear link to technology and in this sense Marx can also be seen as an early philosopher of technology. More so than Kapp, Marx did consider political and ethical questions in relation to technology. However, for Marx too, technology itself is not the problem, but the fact that not everybody benefits from it. Therefore, the mediation figure of *technology as the means for perfecting the human being* finds its complement in the ethical concern of *the need to overcome scarcity and unequal distribution of technology*.

To this day, scarcity and fair distribution remain important themes in the ethics of technology. Think of questions like: Who can and who cannot benefit from expensive medical research and treatment, or, who has access to ICTs and who has not, due to lack of finance or skill? However, next to the accessibility of technology, another theme gained prominence, namely: technology itself may be not so miraculous, but dangerous.

### Limits to the Rule of Technology

About the same time (mid twentieth century) when Canguilhem showed how for the early philosopher of technology Kapp a merger between technology and human existence was only natural and hardly morally problematic, other philosophers began to assert that limits should be set to technological development. The dominant, general conception of technology reversed from optimistic, utopian, to often very pessimistic, dystopian. Unimagined side-effects and downsides of technical progress appeared: the atomic bomb, environmental crisis, social problems related to mass production and consumption, and bureaucracy gone out of hand. Technology was no longer a marginal theme in philosophy but prominent philosophers devoted attention to it and these critiques remain referential until today. This second stage of philosophy of technology was therefore called “classical philosophy of technology” (Achterhuis 2001).

The classical philosophers of technology discovered and conceptualized the dangers of the accumulation of technologies. Ellul (1964) argued that modern technology had become ‘autonomous’ at the expense of the autonomy of humans. Heidegger (1977) believed that the technical way of thinking had come to determine how humans relate to the world: they see the world as a stock of resources for humans to use and manipulate. The experiences with rapidly spreading technology apparently turned around human thinking about technology. The relatively untroubled early reflections made way for an analysis that completely centered on the dangers of technology. Because technology imposes its essence upon man, technology was no longer seen as a condition for further perfection of humans, but as an obstacle to a proper human way of life. The dominant figure of technical mediation is that *technology accumulates into a system which takes command of man*.

Instead of Kapp’s approach of understanding technology, now an ethical perspective came to prevail. In the case of Ellul and Heidegger it seems that the domination by technology was deemed so pervasive that a way out was hardly feasible. Yet, in general, ethics of technology at that time was devoted to the reinforcement of resistance. One example is the thesis by Jürgen Habermas that the ‘lifeworld’ must be protected against ‘colonization’ by the ‘system’ (Habermas 1987). The lifeworld is the sphere where human communication forms the organizational basis. In the sphere of the system economic exchange, institutional procedures, and technology are the structuring principle. Though useful in its proper sphere, a reduction of our whole reality to system characteristics would mean a great threat. Another example is how Hans Jonas (1984) emphasized the need to set limits to the rush of the technical system. The technical era requires a new ‘categorical imperative’ (unconditional prescription, after Kant). For this Jonas wanted to rely on the ‘precautionary principle’: technologies should not be applied until it has been proven that they do not endanger the survival of humanity. To conclude, the task that ethics took on was limiting the rule of the technical system.

In this second stage of the philosophy of technology, the central figure of technical mediation is that technology is accumulating into a system that takes command. This is answered by the ethical concern to *set limits* or *re*-*humanize technology*. Unlike in the earlier ethics of overcoming scarcity of technology, where technology was an unproblematic precondition for human development, now technology itself is considered to be dangerous. Limiting the further development of technology remains an important motif in the ethics of technology to this day. But at the same time the belief that clear criteria exist to demarcate where technology becomes ‘oppressive’ has faded. Also, the panic that many classic philosophers expressed, following the suspicion that often limits have already been exceeded for long and by far, has become less intense. A new theme that has come to the fore is that, for better or worse, humans have become hybrids with technology.

### Hybrids for Better or Worse

The third phase in the philosophy that I want to address is characterized by what has been called the ‘empirical turn’ (Achterhuis 2001). Since the 1970s, philosophers of technology attempted to escape what they now considered to be overly abstract and univocal views on technology of thinkers like Heidegger and Ellul. Some, such as Andrew Feenberg (2002), sought to retain the serious concerns of classical philosophy of technology, but reformulated in a less rigid form, and attempted to elaborate ideas about democratic control of technological development in more concrete and applicable ways. Others, like Don Ihde, Donna Haraway and Bruno Latour, almost seemed to bluntly ridicule classical philosophy of technology. They argued that the human mode of existence cannot be understood in opposition to technology, but only as fundamentally intertwined with it. Haraway (1985) uses the image of the ‘cyborg’, while Latour (1993) argues that humans and things do not exist without each other, but are always ‘hybrids’. We cannot and need not save any original human sphere; but what we can do, according to Ihde (1990), is describe the different kinds of human-technology relations. The figure that *we are hybrids*—*for better or worse* captures such understandings of technical mediation.

Notably Latour has related mediation to ethics with his descriptions of how everyday technologies mediate human behavior. In one of his typical examples, Latour observes how a hotel key with a heavy fob (now largely replaced by access cards) assures that hotel guests do not take the keys with them, but leave them at the hotel desk (Latour 1992). Latour then comments that obedient behavior does not result from an increased sense of moral duty but from the mediation of behavior by a product. Obedience has been ‘delegated’ from human moral consciousness to a thing. Latour further probes that this does not mean that technology overrules morality; to the contrary, he presented his discovery as the ‘missing mass of morality’ (Latour 1992). The new insight would resolve the alleged problem of the decline of morality in our postmodern times. One only needs to understand that behavior always results from the interplay of user intentions and interference by behavior-mediating products.

This statement by Latour, however fascinating and inspiring, was not met with unanimous acclaim. As mentioned in the introduction, Langdon Winner (1993) regretted that critical inspiration was lost, and Huijer and Smits (2010) see the end of ethics unless new moral vocabularies are further developed. Verbeek (2011) and his project of theoretically developing Latour’s probing assertion that action and morality are not reserved for humans but belong to things met with similar critique. Would not such a symmetry come down to the suffocation of ethics and the surrender to the power of technology? Or, if one keeps to the symmetry, what about the confusing consequences, namely that things should also be considered as moral agents, with rights, responsibilities and susceptible to moral appraisal and blame? (Kroes 2012). ‘Nothing is gained but much is lost’ by the way Verbeek confuses and mixes up the different statuses of objects and subjects, assert Illies and Meijers (2009, 425). Martin Peterson goes as far as to assert that Verbeek’s views are ‘either false or misleading’ (Selinger et al. 2014, 303).

In a way, empirical philosophy of technology has returned to the perspective of Canguilhem, who asserted that—in Kapp’s approach—an ongoing merger of humans and technology is only natural. This is in line with my view that technical mediation, as a theme, has been important throughout history. What has changed is rather the ethical evaluation and specific conception of mediation. The ethical problem that Canguilhem postponed came to dominate the debate afterwards and took the form of an attempt to avert hybridization. Contemporary practice-oriented philosophers again emphasize the human-technology merger, and advance detailed, empirical studies on the multiple types of impact of technology. Hybridity is unescapable and the meaning is today considered *ambivalent*, not fitting the rather coarse figures of utopian and dystopian technology.

## Ethics in Times of Technical Mediation

What is the suitable response of ethics to the merger of humans and technology? Can an approach centered around mediation also satisfy the wish of including ethical concerns about the influence of technology into the analysis? Does the empirical turn in research on technology, which describes more than it criticizes how different technologies necessarily mediate people’s lives, mean the end of ethics? Or, can ethics renew itself and find a method and a vocabulary to analyze the interference of technical products in human action in ethical terms as well? A technical mediation theory that rejects the dystopian fear of technology and insists that technical mediation is all around and inescapable does easily fulfill this wish. A denial or attempt to ward off hybridization is indeed infeasible. However, is there a way to avoid that this leads to passivity, indifference or even active approval of what is deemed inescapable anyway?

What can be an appropriate answer to the conception of technical mediation rooted in the idea that we are hybrids? How to elaborate the ambivalence of the impact of technology, in between the utopian embracing and dystopian fear of an ongoing merger? An appropriate answer in general terms seems to go in the direction of *caring for the quality of the interactions and fusions with technology*. This implies an approach that is not only theoretical but also practical. It concerns coping with the technical conditions of our existence as part of the art of living. It is therefore that the work of Michel Foucault on the history of ethics as art of living provides starting points for further elaboration of these issues.

### Foucault’s Ethics as Art of Living

In Foucault’s work there was a remarkable shift of perspective from the study of ‘disciplinary power’ to an interest in ethics as ‘care of the self’. Most of his career, Foucault affirmed that the freedom that modern man believes in is illusory, and that the ‘subject’ is in fact the result of disciplinary practices characteristic of modern society (Foucault 1977). Modernization is accompanied by subjection of people to ever more procedures and detailed surveillance. All in all, modern society looks like a big ‘Panopticon’ (circular dome prison, after Bentham). Foucault’s critique of disciplinary power clearly resembles the analysis of classical philosophy of technology that all technology accumulates into a dominating system.

Foucault’s work on disciplinary power reads like a dramatic revelation of the impotence of ethics. In his later work Foucault approaches individuals no longer as mere victims of power, but gets much more interested in how people themselves cope with external influences on them. As part of an extensive research into the history of sexual ethics, Foucault studied ancient Greek and Latin texts. He discovered that ancient ethics was exactly about the efforts and exercises that everyone should carry out to make himself a virtuous person, to pursue a successful life, to stylize one’s own existence. Foucault’s perspective thus changed from a critique of disciplinary power subjecting people towards practical arts of living whereby people govern and fashion themselves (Foucault 1990, 1992, 2000b). Foucault claims that the ancient arts of living contain elements that are important for a renewal of ethics today. I will very briefly discuss two elements that I consider of central importance for ethics in relation to technical mediation: the conception of human freedom and the notion of care of the self.

Foucault opposes the ancient arts of living to modern code-based ethics. In the ethics of the Christian and the secular modern era humans are conceived of as free beings called to obey moral laws. Ethics became almost identified with rationally explicating the foundation of law, and ‘freedom’ was invariably postulated as a necessary condition. In the ancient arts of living both the problem of freedom and of law received less attention. This inspired Foucault to formulate an alternative conception of human freedom. Freedom is not a state of independence from external influences, but an experience that humans achieve through actively coping with circumstances. This conception of freedom is in line with what Foucault thought to be the purpose of the arts of living in antiquity, namely the striving for active mastery over one’s own life.

Whereas the ancient arts of living put less emphasis on the force of the law, there was more attention to what was called the care of the self. According to Foucault, the free subject is not a precondition for ethics, but any experience of being a subject (the first person perspective of a desire for and ability of agency) consists of active exercises to get a grip on one’s own life. Conducting oneself, being a subject, requires practice and effort. Foucault calls such activities ‘practices of the self’ or ‘technologies of the self’ (technology here in the sense of method, skill). Examples from antiquity are the keeping of diaries, analyzing dreams, physical exercises, dieting, and maintaining friendship with a mentor for counseling.

### Care for the Quality of Our Interactions and Fusions with Technology

In his late work on ethics Foucault hardly returned to the theme of technology. Still, the recombination of the themes of technical mediation and care of the self seems promising for today’s ethics of technology. Firstly, Foucault’s conception of freedom as an experience of mastery helps to clarify how the impact of technology does not necessarily negate human freedom. Every experience of subjectivity has long been intensively mediated by various technologies. New technologies contribute to the coming about of new forms of subjectivity. To exercise freedom is not the opposite of being influenced by technology, but consists in coping with these influences.

Secondly, the concept of self-care is useful. It helps to see how people throughout history have actively worked on themselves to constitute their subjectivity, gained in interaction with the influences of the products they used. An ethics that likes to preserve a clear separation between humans and technology often finds itself watching helplessly how all kinds of technologies do get integrated in people’s existence. Exactly those processes of technology accommodation can be studied very well from the perspective of an ethics of care of the self. What are the considerations and processes at play when people integrate partly dubious technologies into their lives? How do people get ‘used’? How do they manage to adjust technologies to their own ends, so that they become embedded in their lives in a meaningful way?

The care of the self as an approach in ethics thus offers an alternative perspective for the ethical analysis of the social effects of technology. Instead of guarding an assumed frontier between where technology still respects human freedom and where it becomes intrusive, ethics takes on research into the specific forms of interaction and fusion that technologies allow for. Such an analysis combines an exploration of the effects of technical mediation with research into the user’s activities of coping with these effects in their lives. This approach in ethics is about the transformation of one’s existence by the engagement with (new) technologies.

## Technical Mediation Is All Around/Not All There Is

So, taking all the strands that I developed together, can we understand the confusion and contestation directed at Latour and Verbeek from the side of moral and critical philosophy? In everyday language, this may be, simply, because their approaches centered around the notion of technical mediation make the impression of being all too optimistic and uncritical about technological developments. On a deeper level this has to do with how the relation between the influences of technology and ethics is understood. Their approaches in the end equate technical mediation with ethics. Technical mediation is all around, mediates our behavior and even our moral outlook. While this is altogether true, one still expects ethics also at the same time to be “about” the impact of technology. In my interpretation and use of Foucault, the ethics of technology means an ongoing “problematization”, or a “critical ontology” of our technically mediated existence. The aim is finding, or forcing, openings to possible transformations of our way of being.

Latour’s analysis of the delegation of action to things is on the one hand a great tool for problematization. It does help to raise awareness of one’s condition. But, on the other hand, this analysis equates the influences of technology with ethics. For, that is of course the literal meaning of the delegation probe: the laws of morality are replaced by the prescriptions of technologies. The same is true for Verbeek, who follows Latour’s idea of delegation, and tries to elaborate the probe into a theory of mediation.

While it is true that Verbeek has adopted Foucault’s art of living to reconcile technical mediation and ethics along the same line as I, there are essential differences. Verbeek does not see technical mediation and the hybridization of our existence as the material for problematization, as I propose, but as literally the replacement for the moral law. His use of Foucault comes down to a repetition of Latour’s delegation. In Verbeek’s way of thinking, technical mediation is the *answer* instead of the *problem* (the issue, the material worth problematizing). And this would imply (strangely and unintendedly I think) that in our times of technical mediation when ethics entails no longer respect for the moral law, the new ethics would be subjection to the rule of technology.

In the meantime, it appears that for Latour the delegation of morality to things was merely a provisory attempt to redefine ethics. For, in his latest book, the extensive and remarkable *An inquiry into modes of existence*, morality is discussed as the “experience of scruples” (Latour 2013, 443). And this experience is about questioning the conditions of one’s existence: things as they are now, could they be different, can they be changed? For Latour, like for Foucault, it turns out, ethics is ultimately about problematizing the conditions of one’s existence aimed at overcoming. Could it be that Verbeek, in adopting the delegation of morality to things, has been an all-too hasty and loyal follower of the earlier Latour?

Ethics of technology has to be about technical mediation, but this should not mean a leap into subjecting ourselves to the effects of technical mediation. It is also a gesture of reflection on our technically mediated self, aimed at possible transformation. In short: while it is true that *technical mediation is all around, technical mediation is not all there is*.

## Footnotes

1 See Chamayou (2007) for a contemporary commentary, accomapanying Kapp's text in French translation by Chamayou (Kapp 2007).
